# Editorial: Respiratory microbiome in health and disease

**DOI:** 10.3389/fcimb.2023.1335337

**Published:** 2023-11-29

**Authors:** Yimin Zhuang, Tao Ding, Jiangchao Zhao, Jianmin Chai

**Affiliations:** ^1^ Guangdong Provincial Key Laboratory of Animal Molecular Design and Precise Breeding, College of Life Science and Engineering, Foshan University, Foshan, China; ^2^ State Key Laboratory of Animal Nutrition, College of Animal Science and Technology, China Agricultural University, Beijing, China; ^3^ Department of Immunology and Microbiology, Zhongshan School of Medicine, Sun Yat-sen University, Guangzhou, China; ^4^ Department of Animal Science, Division of Agriculture, University of Arkansas, Fayetteville, AR, United States

**Keywords:** respiratory microbiome, health, respiratory disease, respiratory tracts, microbe-microbe interaction, microbe-host interaction

Respiratory diseases, such as COVID-19, pneumonia, asthma, chronic obstructive pulmonary disease, and lung cancer, etc., are leading causes of death and disability in the world. With the development of sequencing technology, the critical roles of the respiratory microbiome in health and disease have been understood (Zhao et al., 2012). As we know, a diverse and dynamic community of microbiomes colonizes the inter-surface of the respiratory system. However, compared to the gut ecosystem, fewer studies focus on the respiratory microbiome and its roles in health and disease. Although a changed respiratory microbiome is associated with a specific disease and host inflammation, more studies need to be conducted to broadly investigate the importance of the respiratory microbiome in health and disease.

A total of 15 original studies published in this Research Topic broaden our knowledge of the respiratory microbiome. These studies generally reveal the potential relationship between respiratory microbiome and various diseases, which benefits our understanding of how the airway microbiota maintain respiratory health and resist disease.

In the past years, the coronavirus disease 2019 (COVID-19) epidemic that spread throughout the world has impacted our life and health. Revealing the critical roles of microbiota may contribute to the prevention and treatment the COVID-19. Ferrari et al. found that Shannon’s entropy and the nasopharyngeal bacterial microbiota (BMN) Factor1 were positively associated with serum anti-RBD-IgG antibody maintenance, suggesting that BNM composition may influence the immunological memory against SARS-CoV-2 infections. Li et al. examined the changes in stool and oral microbiota from the same individuals during the pre-pandemic (before March 2020) and early pandemic (May–November 2020) phases and found that stool and saliva microbiota from the pre-pandemic to early pandemic periods largely exhibited ecological stability (especially stool microbiota), with most associations in loss of diversity or changes in composition related to more reported health issues and pandemic-associated worries. Ling et al. assessed longitudinal changes in the upper respiratory microbiome, its association with disease severity, and potential confounders in adult hospitalized patients with COVID-19. Among all covariates, antibiotic treatment had the largest effect on upper airway microbiota. Longitudinal analysis showed that the upper respiratory microbiota alpha and beta diversity was unchanged during hospitalization in the absence of antimicrobial therapy.

Pneumonia, a common lung infection, causes the air sacs, or alveoli, of the lungs to fill up with fluid or pus in one or both lungs. It usually is caused by bacteria, viruses, or fungi. In terms of other diseases, He et al. compared the difference in the lower respiratory tract (LRT) microbiome between patients with hematopoietic stem cell transplantation (HSCT), healthy controls (HC), and patients with community-acquired pneumonia (CAP). The results showed the diversity of the LRT microbiome significantly decreased in patients with post-HSCT pneumonia, and the overall community was different from the CAP and HC groups. At the phylum level, post-HSCT pneumonia samples had a high abundance of Actinobacteria and a relatively low abundance of Bacteroidetes. The same was true for non-survivors compared with survivors in patients with post-HSCT pneumonia. At the genus level, the abundances of *Pseudomonas*, *Acinetobacter*, *Burkholderia*, and *Mycobacterium* were prominent in the pneumonia group after HSCT. On the other hand, gut-associated bacteria, *Enterococcus*, was more abundant in the non-survivors. Some pathways concerning amino acid and lipid metabolism were predicted to be altered in patients with post-HSCT pneumonia. Hu et al. conducted a multi-omics association analysis to detect the interactions between the oropharyngeal microbiome and the metabolome in pediatric patients with influenza A virus pneumonia, and the results indicated that compared to healthy children, children with IAV pneumonia exhibited significant changes in the oropharyngeal macrobiotic structure and significantly lower microbial abundance and diversity. These changes came with significant disturbances in the levels of oropharyngeal metabolites. Intergroup differences were observed in 204 metabolites mapped to 36 metabolic pathways. Significantly higher levels of sphingolipid (sphinganine and phytosphingosine) and propanoate (propionic acid and succinic acid) metabolism were observed in patients with IAV pneumonia than in healthy controls. Using Spearman correlation analysis, correlations between IAV pneumonia-associated discriminatory microbial genera and metabolites were evaluated. The results indicated significant correlations and consistency in variation trends between *Streptococcus* and three sphingolipid metabolites (phytosphingosine, sphinganine, and sphingosine). Besides these three sphingolipid metabolites, the sphinganine-to-sphingosine ratio and the joint analysis of the three metabolites indicated remarkable diagnostic efficacy in children with IAV pneumonia. Xu et al. concluded that metagenomic next-generation sequencing (mNGS) of bronchoalveolar lavage fluid (BALF) improves the sensitivity of pathogen detection and provides guidance in clinical practice for diagnosing lower respiratory tract infections in children. Moreover, the importance of oral microbiota in other respiratory diseases, such as periodontitis, chronic obstructive pulmonary disease (COPD), and comorbid diseases, was classified. Liu et al. found significant differences in the bacterial community and functional characterization of oral microbiota in periodontitis, COPD, and comorbid diseases. Compared to gingival crevicular fluid, subgingival plaque may be more appropriate for reflecting the difference in subgingival microbiota in periodontitis patients with COPD. These results provide a potential path for predicting, screening, and treatment strategies for individuals with periodontitis and COPD.

The respiratory microbiota in animals affected by environmental factors also correlates with respiratory disease (Chai et al., 2022). In addition to human research, publications in this Research Topic also detect the microbial characteristics of bovine respiratory tract. Howe et al., detected the microbial difference between healthy calves and bovine respiratory disease (BRD) calves and found greater variation in microbial diversity in the BRD calves. Consensus approaches-based random forest, DESeq2, and ANCOM-BC2 were successfully applied to identify signature bacteria. Immigration of the microbiota from the upper airways to the lungs has been confirmed in humans (Zhang et al., 2022). In cattle, Zhang et al. found that the microbial connections among the upper and lower airway were observed in beef cattle regardless of geography, although the microbial diversity, structure, and composition in the upper and lower respiratory tract in beef cattle from China, the United States, Canada, and Italy were significantly different. Regarding the spatial dissimilarities among the respiratory niches, the nostril and nasopharynx had a more similar microbiome compared to the lung communities. Additionally, the major bacterial immigration patterns in the bovine respiratory tract were estimated, and some of them were associated with geography.

Except for the associations between the airway microbiota and respiratory disease, gut microbiota interacting with lung disease and health is another hot topic, which might provide a new treatment direction for respiratory disease. Hu et al. found that acute respiratory distress syndrome (ARDS) altered the gut microbiota of the patients. This study confirmed that the *Escherichia–shigella* genus was effective at distinguishing AP-ARDS from AP-nonARDS, which could predict ARDS occurrence in AP patients.

The present Research Topic highlights the tight associations between the respiratory microbiota and disease. It also reveals the microbe–microbe interaction in the respiratory tract, which is influenced by multiple environmental factors. These recent advancements in the field of respiratory microbiome in health and disease of both animals and humans provide insights into how to manipulate respiratory microbiota to improve host health in the future.

## Author contributions

YZ: Investigation, Resources, Writing – original draft. TD: Resources, Supervision, Writing – review & editing. JZ: Funding acquisition, Project administration, Visualization, Writing – review & editing. JC: Funding acquisition, Writing – original draft, Writing – review & editing, Project administration.
